# Observations on the Guinea Worm

**Published:** 1804-11-01

**Authors:** 


### Observations on the Guinea Worm.

Mr. Larrey had an opportunity of observing, when in Egypt, several inflammatory tumours, which in Africa are generally attributed to the presence of a worm, said to have penetrated under the skin, and where it is universally believed, that the ulceration caused by this animal, cannot be healed but by the complete extraction of it. The method therefore adopted for curing that complaint, consists in winding round a small stick, a whitish fragile filament, which is supposed to be the body of the worm.

The

The greatest caution is recommended not to tear it, for if, unfortunately, it should happen to break, this is thought to produce the most serious consequences from the worm penetrating deeper; amputation of the member affected with this disease is supposed to be necessary, and the life of the patient to be often endangered.

The physicians and travellers who have described this disease, by which white men are but seldom attacked, do not agree with respect to the causes of the formation and the origin of this worm. In Egypt it is called worm of Pharaon; in Africa, Guinea-worm; in the West-Indies, Vena Medinensis; and in Jamaica, Colubrilla.

Mr. Larrey thinks that the symptoms attending these tumours, which he considers as a simple furunculus or anthrax, are produced by the operation undertaken for the purpose of extracting the worm, and which are aggravated when the operation fails. He has very attentively examined the nature and form of the whitish filament, but he could not discover the slightest analogy with a worm; and he even convinced himself, by dissection, that this thread is nothing but dead cellular texture, which is drawn into a filament through a hole of the skin, when a small portion of it is seized in order to twist it round a piece of wood; and it is by means of this improper manœuvre, that cylindrical pieces of this cellular texture are obtained long enough to be confounded with a true worm. He has since had many opportunities of ascertaining this assertion; by seizing the cellular texture in a simple furunculus with the pincers, he obtained the same result. Mr. Larrey acknowledges that he is of the same opinion with Dr. Delaborde, who during his residence at Cayenne, after a great number of observations, adopted the same idea.

Mr. Larrey has added to his memoir, some observations on two negroes attacked with the vena medinensis, whom he treated at Cairo. The first, aged nine years, was committed to the care of an Egyptian physician, who immediately began to wind out the supposed worm, whereby the young patient suffered the greatest pain. The furunculus was situated on the malleolus internus, and surrounded with a bluish ring, which made the part appear as if it was becoming gangrenous. Mr. Larrey, however, having cut off the thread as close to the furunculus as possible, applied emollients with saffron on the tumour, and administered to the patient alternately diluents with Peruvian bark. A few days after an abscess had formed itself, which being opened with a bistouri, the patient got better, and was cured in a short time.

The anthrax of the second negro had formed on the foot, and from the point of this ulceration issued a blackish core, which would have been taken for the head of the worm. Mr. Larrey prescribed emollients without touching the supposed worm. The inflammation ran through its periods, and an abscess was formed, which he opened as in the former case. The condensated cellular texture issued with the pus in form of small flakes, and the negro was perfectly cured in a fortnight after he had perceived the complaint.

